# Metal ion-directed solution-phase tailoring: from large-area graphene oxide into nanoscale pieces

**DOI:** 10.1186/1556-276X-8-226

**Published:** 2013-05-14

**Authors:** Xiansong Wang, Peng Huang, Huiyang Liu, Chao Li, Guangxia Shen, Daxiang Cui

**Affiliations:** 1 Department of Bio-Nano-Science and Engineering, Key Laboratory for Thin Film and Microfabrication of Ministry of Education, Institute of Micro-Nano Science and Technology, Shanghai Jiao Tong University, 800 Dongchuan Road, Shanghai 200240, China

**Keywords:** Graphene oxides, Nanoscale graphene oxide pieces, Spontaneous redox reaction, Metal particles

## Abstract

Due to fascinating electronic properties and great potential in various applications, graphene has attracted great interest. Recently, much work have focused on the synthesis of different sizes and properties of graphene or graphene oxides (GOs), for example, graphene nanoribbons, nanosized graphene pieces, and nanosized triangular and hexagonal graphene sheets terminated by zigzag edges. Herein, we have demonstrated a widely available approach to fabricate the nanoscale GO pieces by directly solution-phase cutting a large-area GO sheet into nanoscale pieces via spontaneous redox reactions at room temperature. In this process, GO acts with dual functions as a model and a reducing reagent. With a typical example of silver ions, we have investigated in detail the influence of the reaction time and concentration of metal ions on yield and size of nanoscale GO pieces. Moreover, we also obtain Ag nanoparticle coating on the GO surface. Finally, a possible mechanism is suggested to explain the formation of nanoscale GO pieces.

## Background

Since discovered by Andre Geim and Konstantin Novoselov in 2004 [1], graphene has drawn significant attention to different scientific and technical communities due to its unique electrical, chemical, mechanical, optical, and structural properties [2]. However, large-area graphene remains to be a metallic conductor even at the neutrality point which limits its application in nanoelectronic devices and biological science [3-6]. In addition, for the purpose of drug delivery and biological nanoprobe applications, small-sized graphene or graphene oxides (GOs) can easily be swallowed into organs, tissues, and cells [7]. Recently, quite a lot of researchers have reported about the preparation of graphene ribbons with quantum confinement and edge effect properties by directly tailoring large-area graphene via e-beam lithography [8], hydrogen plasma etching [9], scanning tunneling microscope lithography [10], atomic force microscopy [11], chemical stripping, or catalytic tailoring (Fe, Ni, and Co nanoparticles as catalysts) [12-16]. Usually, the technologies used for synthesis of graphene ribbons mostly must be operated under ultrahigh-vacuum and high-energy conditions. So it is very difficult to produce large quantities of water-soluble graphene pieces. Moreover, these extreme synthetic conditions will be ultimately bound to affect the properties of graphene ribbon. Till now, direct soluble-phase formation of nanoscale graphene or graphene oxide pieces has been rarely involved [17]. Generally, through selecting small-sized graphite as raw materials to control the size of GO during the synthesis of GO through the Hummers procedure, subsequently complicated treatment with strong sonication treatment and stepwise centrifugation at 4,000 to 10,000 rpm, a small-sized GO can be obtained [18]. However, the procedures are quite complex and the yield of nanoscale fragments is also very low.

Herein, we report a widely available approach to prepare the nanoscale GO pieces directly utilizing some oxidizing metallic ions (Ag^+^, Ni^2+^, Co^2+^, etc.) via spontaneous redox reactions to cut a large-area GO sheet into nanoscale pieces at room temperature. With an example of silver ions, we have investigated the influence of the reaction time and concentration of metal ions on size and properties of nanoscale GO pieces. Meanwhile, the corresponding silver nanoparticles can also be obtained. Finally, a possible mechanism is put forward for explaining the formation of nanoscale GO pieces.

## Methods

### Chemicals

All reagents were of analytical grade and purchased from Shanghai Sinopharm Chemical Reagent Co., Ltd. (Shanghai, China). Natural graphite powder (800 mesh) was provided by Beijing Chemical Reagents (Beijing, China). All aqueous solutions were prepared with ultrapure water (18 MΩ cm).

### Preparation of large-area GO

Water-soluble GO was prepared by oxidizing graphite according to a modified Hummers method just as our previous reports [19,20]. Briefly, the graphite powder was first oxidized into graphite oxide using KMnO_4_/H_2_SO_4_, and then the graphite oxide was exfoliated into GO sheets in water under ultrasonication for 1 h, followed by centrifugation at 4,000 rpm for 30 min and dispersion in water. The obtained yellow-brown aqueous suspension of GO was stored at room temperature for further characterization and subsequent reaction.

### Preparation of nanoscale GO pieces

The experiments of cutting large-area GO were carried out as follows: Firstly, 100-mL GO water solution (0.50 mg/mL) was prepared. Homogeneous suspension (20 mL) of GO was mixed with the desired amount of aqueous metallic ion (Ag^+^, Ni^2+^, Co^2+^, etc.) solution (5 mg/mL). Without heating or ultrasonication, the reaction mixtures were kept at room temperature for 48 h. Then the mixtures were centrifuged to remove the nanoparticles and large-scale GO and particle composites at the rate of 8,000 rpm. The upper solution without further purification was detected by atomic force microscopy (AFM), Fourier transform infrared (FTIR) spectroscopy, UV-vision (UV-vis) spectroscopy, and X-ray photoelectron spectroscopy (XPS). In order to investigate the tailoring mechanism, we selected silver ions as a typical example and elaborately investigate the influence of reaction time and concentration of silver ions on the size and properties of nanoscale GO. All experiments were carried out at 25°C ± 2°C.

### Characterization of nanoscale GO

AFM images were obtained on a Nanoscope MultiMode V scanning probe microscopy system (Veeco, Plainview, NY, USA) by tapping-mode imaging. Commercially available AFM cantilever probes with a force constant of approximately 48 N/m and resonance vibration frequency of approximately 330 kHz were used. The scanning rate was usually set at 1 to 1.2 Hz. Freshly cleaved mica with atom-level smoothness was used as the substrates. The samples were coated on the mica surface by spin-coating technology. UV-vis spectra were measured at 20°C with a Shimadzu UV-2450 spectrophotometer equipped with a 10-mm quartz cell (Kyoto, Japan). Zeta potentials were measured with NICOMP 380 ZLS Zeta Potential/Particle Size Analyzer. The XPS measurements were performed on an Axis Ultra DLD XPS (Kratos Analytical, Manchester, UK) using a monochromated Al Kα (1,486.6 eV) source at 15 kV. Scanning electron microscopy (SEM) images were taken on a ZEISS-ULTRA 55 SEM (Oberkochen, Germany) equipped with an X-ray energy-dispersive spectroscope (EDS) at an accelerating voltage of 20 kV (provided in Additional file 1). In addition, the conductive properties of the nanoscale GO film coated on the mica surface were tested using a conductive AFM. The detailed process and results have been given in Additional file 1.

## Results and discussion

### Tailoring large-area GO by different metal ions

**Figure 1:**
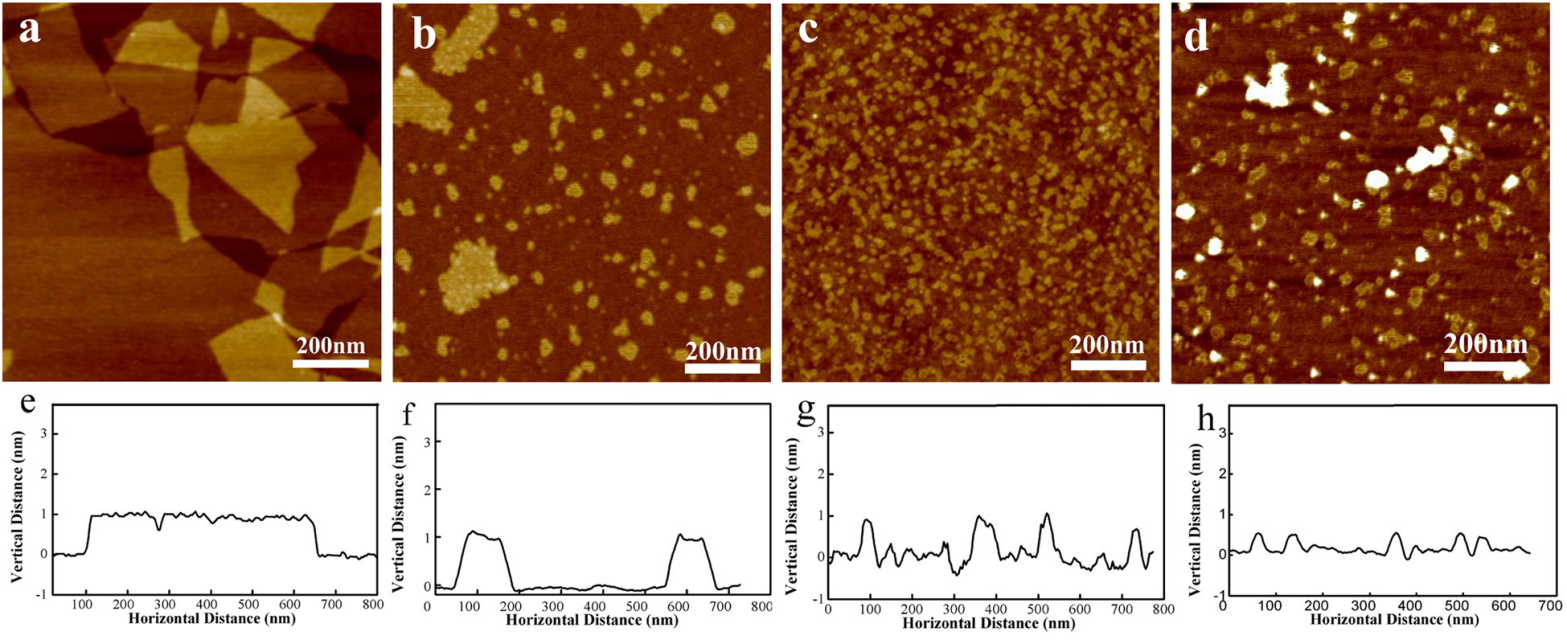
**Tapping-mode AFM images of GO and nanoscale GO pieces. **(**a**) GO, (**b**) Ag^+^, (**c**) Co^2+^, and (**d**) Ni^2+ ^and corresponding profile analysis: (**e**) GO, (**f**) Ag^+^, (**g**) Co^2+^, and (**h**) Ni^2+^.

Graphene oxide is very widely generated using natural graphite powder through the Hummers method. The chemically derived GO is soluble in pure water due to hydrophilic functional groups, e.g., carboxyl, hydroxyl, and epoxide groups on the surface [16,21]. Figure 1a shows the AFM image of GO with atom-level smoothness and the sizes in the range of 1 to 10 μm. The height profile of the AFM image in Figure 1e is approximately 1 nm, which is consistent with the data reported in the literature, indicating the formation of a single-layered GO. Figure 1b,c,d depicts that the nanoscale GO pieces with different sizes were tailored utilizing three kinds of metal ions (Ag^+^, Ni^2+^, Co^2+^), respectively. Corresponding profile analysis of these AFM height images (Figure 1f,g,h) has given heights of approximately 1 nm, which were elementally consistent with the thickness of GO. Similarly, in the addition of Ag^+^ ion system, some nanoparticles have been found to be dispersed in the solution or attached on the GO surfaces similar to what we have reported previously [22]. In our previous work, we mainly focused on the synthesis of silver-GO composites. When testing the samples by AFM, some little pieces were occasionally detected in the high-resolution images, which were neglected as contamination before [22]. Thereafter, in order to investigate the tailoring mechanism, we selected the other weak oxidation of metal ions, such as Ni^2+^ and Co^2+^, and obtained results similar to the information given previously. In addition, XPS data have been provided in Additional file 1: Figure S1.

### Tailoring large-area GO by silver ions

**Figure 2:**
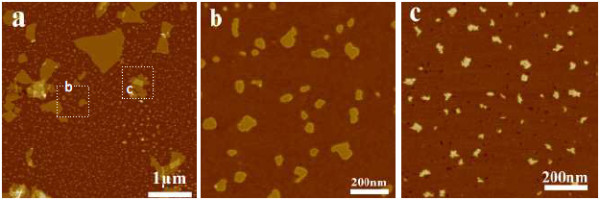
**Tapping-mode AFM image of nanoscale GO pieces using 0.5 mM silver ions for 24 h. a**)nanoscale GO; (**b**) and (**c**) the high- resolution images of the labeled area in (**a**).

For silver ions, a series of systematic experiments have been carried out. In a typical experiment, 0.50 mg/mL of an aqueous GO dispersion (10 mL) was added to 10 mM aqueous AgNO_3_ solution (10 mL). As shown in Figure 2a, the large-area GO has been tailored into small fragments after the reaction was kept for approximately 12 h. TEM image and EDS data were given in Additional file 1: Figure S2. They provide the presence of Ag, C, and O elements, which should be attributed to GO and the silicon substrate. Furthermore, the sizes of the little pieces do not become smaller even if the reaction time is beyond 48 h. Meanwhile, there were two kinds of nanoscale GO existing in the mixture: one is the pure nanoscale GO pieces in Figure 2b, and the other is the silver-GO composite pieces in Figure 2c. In addition, the nanoscale GO film cannot be conductive using C-AFM testing (see Additional file 1: Figure S3).

**Figure 3:**
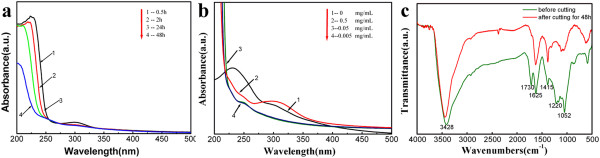
**UV-vis absorption and FTIR spectra of nanoscale GO. **(**a**) UV-vis change with reacting time, (**b**) UV-vis change with adding Ag^+ ^concentration; (**c**) FTIR spectra of nanoscale GO by adding 0.5mM Ag^+ ^after reacting 12h.

Influence of the reaction time on the sizes and properties of nanoscale GO pieces was monitored by UV-vis spectroscopy (Figure 3a). The UV-vis spectra of GO display two characteristic peaks at 230 and 303 nm, corresponding to π → π* transition of aromatic C-C bond and *n* → π* transition of C=O bond, respectively [23]. From Figure 2d, it can be found that the two characteristic peaks of GO red-shift to approximately 250 and approximately 310 nm after adding 0.5 mM Ag^+^ ions into the GO solution for 0.5 h, due to the interaction of GO and silver ions. The peak intensities decayed gradually with prolonged reaction time. Especially the peak intensity in the region approximately 310 nm decreases dramatically after 48 h, providing a first hint that some functional groups in GO may decrease [24]. Similar results can be further achieved by changing the concentration of Ag^+^ ions in Figure 3b. We can find that there is a distinct difference in wavelengths and intensities of the characteristic peaks of GO with the different concentrations of Ag^+^ ions in the system after approximately 24 h. At lower concentration, the signal at 310 nm nearly disappears and that at 250 nm becomes distinct, which may mean that the Ag^+^ ions preferentially attack the sites of *sp*^3^ carbon clusters or defective regions on the basal planes and partially restore the *sp*^2^ carbon framework. When a higher proportion of Ag^+^ ions (5 or 0.5 mM) are added into the reaction system, the peak intensity (at approximately 310 nm) of GO seems to be obvious and accompanies a larger red shift with increasing Ag^+^ ion concentration, gradually close to 360 nm which is for silver plasmon absorption bands [24]. It can be explained that the number of silver nanoparticles fabricated on the GO surface or solution becomes large with the increasing proportion of Ag^+^ ions in the mixture, which also provides more change for the interaction of Ag nanoparticles and GO. At the same time, we also find that even if the Ag^+^ concentration is increased to 5 mM, there still exists some nanoscale GO with smooth edges in the mixture.

FTIR spectroscopy has been considered as another powerful tool to analyze surface chemical group changes of GO. As shown in Figure 3c, the characteristic peaks of GO (green line) displayed the C=O stretching vibration peak at 1,730 cm^-1^, the vibration and deformation peaks of O-H groups at 3,428 and 1,415 cm^-1^, respectively, the C-O (epoxy groups) stretching vibration peak at 1,220 cm^-1^, and the C-O (alkoxy groups) stretching peak at 1,052 cm^-1^[25]. After the reaction is conducted for 48 h (red line), the intensities of the FTIR peaks corresponding to the C-O (epoxide groups) stretching vibration peak at 1,220 cm^-1^ disappeared nearly, the C=O stretching vibration peak at 1,730 cm^-1^ decreased dramatically, and the vibration and deformation peaks of O-H groups at 3,428 and 1,415 cm^-1^, respectively, and the C-O (alkoxy groups) stretching peak at 1,052 cm^-1^ increased slightly. These results further confirmed that some active functionalities (epoxide groups) in GO have been removed.

### The mechanisms of tailoring GO

**Figure 4:**
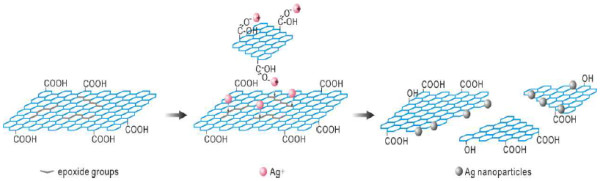
Schematic diagram of tailoring mechanism through solution-phase redox reaction by adding metal ions into solution.

Since the appearance of GO, the determination of GO structure has been challenging because of its nonstoichiometric chemical composition, which depends on the synthesis method and the degree of reduction, and the oxygen functional groups in GO have been identified by various kinds of techniques. It is generally agreed that oxygen is present in GO mostly in the form of hydroxyl and epoxide groups on the basal plane, whereas smaller amounts of carboxyl, carbonyl, phenol, lactone, and quinone are present primarily at the sheet edges. The existence of the chemical groups confers new properties on GO such as the perfect monodispersity in water and weak reducibility. Based on the above facts and our experimental results, a probable mechanism is put forward as given in the schematic diagram (Figure 4). Firstly, part of Ag^+^ ions is preferentially absorbed on the sites of carboxylic groups at the edges of GO by the electrostatic interaction. Then Ag^+^ ions bonded on GO or freely dispersing in the solution further encounter the reducing groups (e.g., epoxy groups) on the basal plane of other GO sheets. Thus, Ag^+^ ions themselves are reduced to Ag and then generate Ag nanoparticles; meanwhile, the carbon-carbon skeleton is broken which directly leads to the cutting of GO into little pieces.

**Figure 5:**
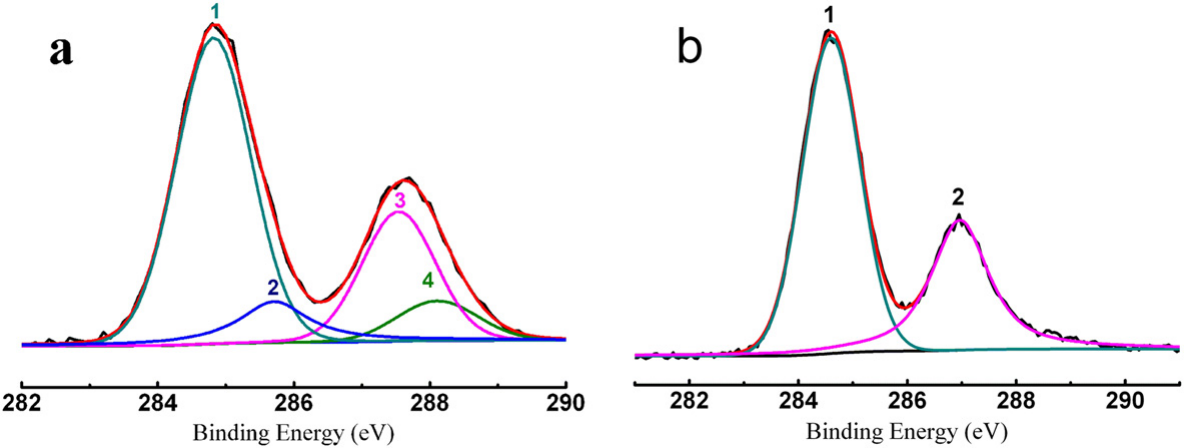
**C**_**1s **_**XPS of GO and nanoscale GO sheets. **(**a**) GO before cutting reaction; (**b**) nanoscale GO after cutting reaction. The peaks 1, 2, 3, and 4 correspond to C=C/C-C in aromatic rings, C-O (epoxy and alkoxy), C=O, and COOH groups, respectively.

Although the feasibility conclusion has been verified through analysis results of UV-vis and FTIR data, we also elaborately investigated the chemical state change of carbon in GO by XPS technology. Figure 5a shows the C_1*s*_ XPS of GO sheets. There are four different peaks detected that centered at 284.5, 288.4, 293.8, and 296.6 eV, corresponding to C=C/C-C in aromatic rings, C-O (epoxide and alkoxy), C=O, and COOH groups, respectively [26]. After adding Ag^+^ ions into solution for 48 h, the distinct changes of C_1*s*_ XPS are detected in Figure 5b. The intensities of C_1*s*_ peaks of the carbons binding to oxygen, especially the peak of C-O (epoxide and alkoxy), decreased dramatically, which reveals that most epoxide groups reacted with Ag^+^ ions. Moreover, the diameters and charges of metal ions may have great influence on the sizes and properties of nanoscale GO which will be further confirmed by subsequent work.

## Conclusions

In summary, we have demonstrated a very simple strategy to obtain nanoscale GO pieces using metal ions as oxidation reagent at mild condition. Without being heated or treated ultrasonically, two kinds of nanoscale GO pieces: GO pieces and nanoparticle-coated GO piece composites, are obtained. Based on systematic investigations of nanoscale GO piece formation by the addition of Ag^+^ ions as a tailoring reagent, a probable mechanism is suggested to explain the formation of nanoscale GO pieces, which can be mainly attributed to interaction of metal ions (Ag^+^, Co^2+^, Ni^2+^, etc.) with the reducing groups (e.g., epoxy groups) on the basal plane of other GO sheets. Obviously, in this progress a large-scale GO acts with dual functions, as a reducing reagent and a nucleation site of metal or metal oxide nanoparticles. This work provides a good way or chance to fabricate nanoscale GO pieces and GO composites in water solution and more widely apply in nanoelectronic devices, biosensors, and biomedicine.

## Competing interests

The authors declare that they have no competing interests.

## Authors’ contributions

XW and PH participated in the preparation of GOs and GO nanosheets. HL and CL participated in the characterization of GOs and GO nanosheets. GS and DC participated in the design and coordination of this study. All authors read and approved the final manuscript.

## Supplementary Material

Additional file 1 **Supporting information. **The file contains **Figures S1**, **S2**, and **S3** and a discussion of the conductive testing by conductive atomic force microscopy. Click here for file
