# Supplementary material for: Metal ion-directed solution-phase tailoring: from large-area graphene oxide into nanoscale pieces
Source: Nanoscale Res Lett. 2013 May 14;8(1):226. doi: 10.1186/1556-276X-8-226 (PMC3665674; doi:10.1186/1556-276X-8-226)
Supplement: Additional file 1 — Supporting information. The file contains Figures S1, S2, and S3 and a discussion of the conductive testing by conductive atomic force microscopy. [file 1556-276X-8-226-S1.pdf]

Additional file 1

Supplementary information

Metal ion-directed solution-phase tailoring: from large-area graphene oxide into nanoscale pieces

Xiansong Wang, Peng Huang, Huiyang Liu, Chao Li, Guangxia Shen,* and Daxiang Cui*

Key laboratory for thin film and microfabrication of the ministry of education,Research Institute of Micro/Nano Science and Technology, Shanghai Jiao Tong University,Shanghai, 200240, P. R. China

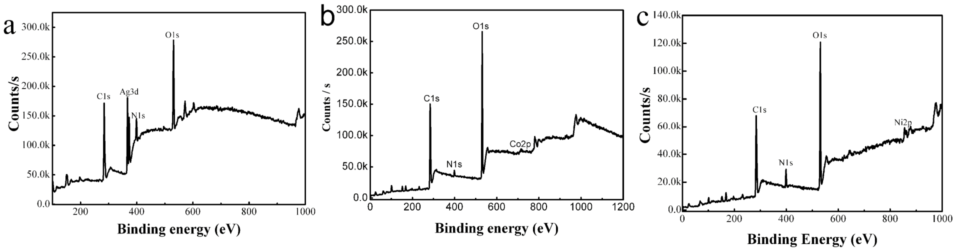

Figure S1 XPS survey of nanoparticles and GO coatings adding into different metal ions for 48h. a) Ag^{+}; b) Co^{2+}; c) Ni^{2+}.

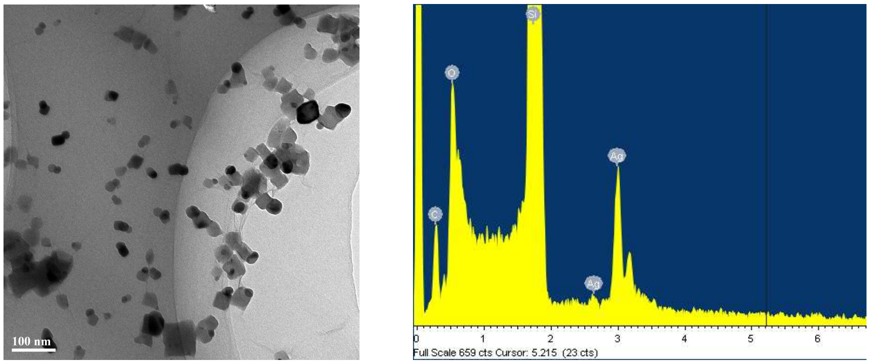

Figure S2 TEM image (a) and EDS analysis (b) of Ag nanoparticles coated on the GO surface.

Conductive testing by conductive atomic force microscopy (CAFM) :

I-V test were carried out by CAFM. Firstly, the Ag nanoparticles and GO mixture solution were coated on the freshly cleaved mica surface. The samples were dried at room temperature for 12h. And then conductive Ag glue was printed on the side of the sample and interface at the AFM slice and samples. The samples were heated at 60°C for 2h in vacuum furnace. A commercial SCM-PIC conductive AFM tip was used. The inset of Figure S2 shows the schematically a two-electrode system based on C-AFM) used for the electrical conductivity measurements of the composite coatings. I-V curve are given in Figure S2. It is confirmed that the composite coating cannot be conductive.

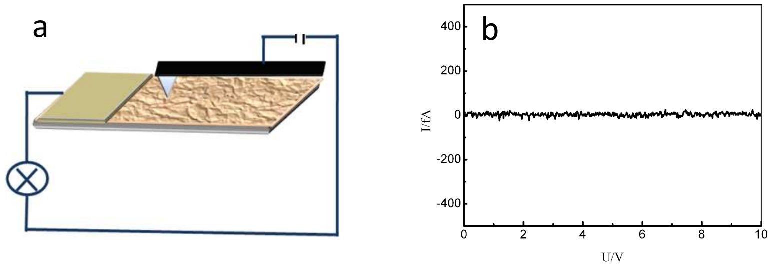

Figure S3. a) a schematically two-electrode system based on conductive atomic force microscopy (CAFM); b)I-V curve of Ag nanoparticles and GO composite coatings used for the electrical conductivity measurements of the composite coatings
